# Dr. G. V. Black’s Conclusions Reviewed

**Published:** 1897-12

**Authors:** B. Holly Smith

**Affiliations:** Baltimore, Md.


### DR. G. V. BLACK'S CONCLUSIONS REVIEWED.[1]

BY DR. B. HOLLY SMITH, BALTIMORE, MD.

SINCE the publication of Dr. Black's conclusions in reference to caries, and the description of the equipment and laborious experiments which seemed necessary in order to partially investigate the subject, it has been apparent to the casual observer who has a disposition to be honest that an element of unfairness must be manifested by any one who would rush into a discussion of the subject without greater thought and investigation than the average practitioner has opportunity for.

---

[1] Read before The New York Institute of Stomatology, October 5, 1897.

From several sources suggestions, resolutions, and appropriations have come for the encouragement of similar work on the further prosecution of Dr. Black's labors. This, of itself, is an evidence that the members of the dental profession have confidence in the value of the work already done, and appreciate the fact that if definite conclusions are to be reached it must be through men who have special talent, opportunity, and equipment. There is, however, a phase of the question through whose discussion we may reasonably hope to aid the investigators, and that is the clinical aspect. The announcement has been made that Dr. Black's theory is entirely at variance with commonly accepted opinions based on clinical experience, but it seems to me not very hard to harmonize Dr. Black's theory with clinical observations. The inharmony at first apparent may result not so much from any fallacious deductions of Dr. Black's as from the long-standing misconceptions of those who have written and thought upon this subject.

The theory that disintegration and loss of teeth by child-bearing women is due to an extra demand for lime salts is unreasonable and untenable, since the process thus becomes a physiological one rather than pathological, while in the light of Dr. Black's discoveries and formulations the altered and perverted secretions known to exist during pregnancy become prime factors in a pathological process which is not arrested by any degree of density of tooth-structure.

Again, the sudden deterioration of teeth which for many years have been free from caries has been heretofore almost without reasonable explanation. A case in my own practice, which is possibly no more typical than has been observed by every practitioner, I will cite. Captain L., with a record of twenty years' police service, upon retirement was in possession of a perfect denture; so far as known no caries existed. He accepted in-door employment of a sedentary character, and three years later had between twenty and twenty-five cavities filled. In the next five years he was almost constantly under my care, visiting me every few months, nevertheless lost the pulps of several teeth; two were extracted and some crowned. A few weeks would often suffice for caries to make its way to the pulp of a tooth, which had the appearance of great density, and whose history would indicate it to be dense.

Since the promulgation of Dr. Black's theory this mouth has been painstakingly treated with antacids and antiseptics, with the results that no teeth have been lost and three stoppings have been introduced during the past year.

Surely there is a vista of hope opening out in the direction of this new theory, which we expect to see broaden into a plain and easy method of salvation for sick and sore dental organs.

